# Monkeypox virus: past and present

**DOI:** 10.1007/s12519-022-00618-1

**Published:** 2022-10-10

**Authors:** Ya-Mei Dou, Hang Yuan, Hou-Wen Tian

**Affiliations:** NHC Key Laboratory of Medical Virology and Viral Disease, Chinese Center for Disease Control and Prevention, National Institute for Viral Disease Control and Prevention, 155 Changbai Road, ChangPing District, Beijing, 102206 China

**Keywords:** Monkeypox, Orthopoxvirus, Smallpox, Zoonotic

## Abstract

**Background:**

The objective of this paper is to analyze the current status of monkeypox worldwide. In the face of this public health threat, our purpose is to elucidate the clinical characteristics and epidemiology of monkeypox, the developmental progress of monkeypox-related drugs and the vaccines available.

**Data sources:**

The literature review was performed in databases including PubMed, Science Direct and Google Scholar up to July 2022.

**Results:**

Since May 2022, the World Health Organization has reported more than 45,000 confirmed cases from 92 nonendemic countries, including nine deaths. Although some women and children have been infected so far, most cases have occurred among men who have sex with other men, especially those with multiple sexual partners or anonymous sex.

**Conclusions:**

Pediatric monkeypox infection has been associated with a higher likelihood of severe illness and mortality than in adults. Severe monkeypox illness in pediatrics often requires adjunctive antiviral therapy. It is crucial for all countries to establish sound monitoring and testing systems and be prepared with emergency preparedness.

## Introduction

Monkeypox is a rare, sporadic, smallpox-like zoonotic infectious disease caused by monkeypox virus, an orthopoxvirus genus of the *Poxviridae* family [1, 2]. The disease frequently occurs in Central and West African countries, especially the Democratic Republic of Congo (DRC), where it is considered endemic [3, 4]. Early research indicates that human infection with monkeypox virus occurs most commonly in the 5- to 9-year-old age group, particularly in small villages where the children hunt and eat squirrels and other small mammals [5]. In the last few years, the United Kingdom, the United States, Singapore and Israel have reported the existence of imported cases in individuals with an African travel history [6–8]. Monkeypox has recently grown to be a global concern, as the World Health Organization reported over 45,000 confirmed and suspected cases (as of August 29, 2022) in more than 90 countries in Europe, the Americas, the Eastern Mediterranean, the Western Pacific and Southeast Asia [9, 10]. Moreover, the number of cases is expected to continuously increase. Significantly different from the past, the vast majority of cases recently reported have no established travel links with endemic areas, involve community transmission and include a small number of women and children [most of the original cases occurred in men who have sex with other men (MSM)] [11], indicating that cases in children may be more frequently reported in the future.

The uncertainty of monkeypox epidemic control and the risk of transmission at the social level have increased the possibility of cross-border spread and onward transmission of monkeypox disease. Therefore, this manuscript not only briefly introduces the family *Poxviridae* but also reviews the clinical manifestations, epidemiology, drug treatment and vaccine application, prevention and control strategies of monkeypox in detail.

## Family *Poxviridae*

The virus that causes monkeypox, monkeypox virus (MPXV), was first discovered in 1958 as the source of infection that caused an outbreak of pustular rash illness in cynomolgus monkeys shipped from Africa to Copenhagen, Denmark, for research purposes. Hence, the name “monkeypox” [12–15]. Later, in 1970, human monkeypox was discovered in an infant who had presented with smallpox-like eruptions in the DRC [16]. Two main clades of human MPXV have been identified: the Central African strains and the West African (WA) strains, the former being more virulent in nonhuman primates. Evidence indicates that the lethality rates are 10.6% and 3.6% for the strains, respectively [17, 18]. MPXV is a double-stranded DNA virus of the orthopoxvirus (OPXV) genus of the family *Poxviridae* [19, 20]. *Poxviridae* are classified into two subfamilies: *Entomopoxvirinae* and *Chordopoxvirinae* [21]. There are four genera of the subfamily *Chordopoxvirinae* that can induce human diseases. Among them, viruses from the genera orthopoxvirus, parapoxvirus and yatapoxvirus harbor zoonotic potential [22]. The genus OPXV mainly contains viruses that infect humans: monkeypox virus, cowpox virus, vaccinia virus, and smallpox virus. According to previous studies, gene homology within OPXV can reach 90% based on immune cross protection and cross reactivity [23]. The cross protection allows individuals who had been infected by any member of the genus to be protected against an infection with another virus from the same genus. This is the scientific basis behind Edward Jenner’s cowpox inoculations and vaccinia virus Tian Tan strain isolated in China protecting against variola virus (VARV) [24–26].

## Clinical presentation

In most cases, monkeypox is a rare but potentially serious viral illness that usually begins with a flu-like illness and swollen lymph nodes and progresses to a widespread rash on the face and body [27]. Monkeypox and smallpox have similar appearance, distribution and pathological progression; however, monkeypox is often less severe [2, 14]. The severity of disease depends on the patient’s age and comorbidities, and case fatality in monkeypox has been reported up to 15%, with younger children being at highest risk [28]. In 1987, pronounced lymphadenopathy was identified as the only clinical sign differentiating monkeypox from smallpox and chickenpox (varicella) [29].

In general, adults or children infected with monkeypox will experience three stages: incubation, prodromal, and rash periods. The incubation period of monkeypox is usually 7–14 days, but it can be longer (5–21 days), with symptoms and signs lasting two to five weeks [30]. Notably, using vaccination after exposure to monkeypox (within four days) given the long incubation period (versus COVID, where the incubation period is shorter) is essential for children and adults [31]. The following prodromal features include fever, muscle aches, headache, backache, sore throat and swollen lymph nodes, followed by a broad, well-circumscribed rash typical of an eccentric pattern. Within one to three days (sometimes longer) after the patient develops fever, the patient develops skin rash, which usually starts from the face and then spreads to other parts of the body. These rashes then go through five stages: macular stage, papule phase, vesicular phase, pustular phase, and finally enter the scab phase [32, 33]. Patients with monkeypox have traditionally been considered infectious until all the lesions have crusted.

However, since May 2022, monkeypox cases in many countries have been atypical in that the rash is starting at genital areas [34]. Available data show that 98.2% of monkeypox cases (22, 548/22954) are male, and the median age is 36 years. At present, this symptom occurs only in adults [10]. It must be noted that although the clinical manifestations of monkeypox are milder than those of smallpox, the disease can be fatal, with mortality rates ranging from 1% to 10% [1, 13, 35, 36]. Newborns, children and immunodeficient patients may face more acute monkeypox symptoms and risk of death. Moreover, severe monkeypox cases can develop complications, including skin infections, pneumonia, confusion, and eye infections, that can lead to vision loss [13]. These complications often occur in children or individuals with other comorbidities and are common in Central Africa. For example, in 2016, a case of a 4-year-old boy was reported in the DRC. The boy presented with the typical clinical manifestations described above, including low-grade fever (37.9 ℃), rhinitis, conjunctivitis, severe left-sided cervical lymphadenitis, and a nonitchy vesiculopapular rash on admission. In the following two to five days, he experienced fever up to 38.5 ℃, and the rash began to spread to cover his entire body surface, including palms, foot soles, and mucous membranes, the latter resulting in severe and painful stomatitis. Although the patient received supportive care, the skin and oral features became progressively worse, and the child died on day 12 post admission [37]. In contrast, the first case of monkeypox outside Africa occurred in the United States in 2003: a 6-year-old girl was intubated and mechanically ventilated due to encephalitis, and a 10-year-old girl had tracheal airway damage secondary to cervical lymphadenopathy and a large retropharyngeal abscess. Although one in five pediatric patients developed serious complications in the United States, no deaths occurred due to intensive medical intervention [33]. In general, the clinical symptoms of monkeypox infection in children outside Africa are milder than those in central and West African countries. The vast majority of monkeypox infections in Africa occur in children and have a very high mortality rate, which is likely an unfortunate consequence of the lack of access to medical services. In addition, different nutrition habits, health conditions and economic education levels can make a significant difference in the risk and symptoms of monkeypox infection [37, 38].

It is challenging to distinguish monkeypox, chickenpox and smallpox only according to these clinical manifestations. Reasonable laboratory diagnostic methods, such as nucleic acid amplification tests, including real-time or conventional polymerase chain reactions, to detect the unique sequence of viral DNA are also necessary for the confirmation of MPXV infection [39–41].

## Epidemiology

Monkeypox is a viral zoonosis (a virus transmitted to humans from animals) with symptoms very similar to those seen in the past in smallpox patients, although it is clinically less severe [42]. Monkeypox has not attracted much attention since its discovery 65 years ago. In 1970, in Central Africa, a 9-month-old child from Zaire (now the DRC) became infected with MPXV, the first human infection reported in history [43–45]. The number of human monkeypox cases has been on the rise since the 1970s, with the most dramatic increases occurring in the DRC. The median age at presentation has increased from 4 (1970s) to 21 years (2010–2019) [46].

## Regional characteristics of monkeypox

Ever since its discovery, the disease has been endemic to Central and West Africa, with sporadic, intermittent cases of monkeypox transmitted from local wildlife reported among humans. Monkeypox disease in Central Africa has clearly been proven to be transmissible between humans and can result in death. The case fatality rate is on average approximately 10% in nonvaccinated individuals. However, the number of reported human monkeypox diseases in West Africa is limited, which is comparably less severe and demonstrates less human-to-human transmission than that in Central Africa [47, 48]. In 2003, monkeypox broke out in the United States. At that time, the monkeypox epidemic was relatively large, and it spread to six states, including Wisconsin, Illinois, Indiana, Kansas, Missouri and Ohio [49]. A follow-up investigation at the time found that the monkeypox outbreak may have been linked to the importation of rodents, resulting in 47 confirmed or probable cases. Although the clinical features of most patients were milder than typically seen in Africa, nine patients were hospitalized as inpatients, and five patients were defined as being severely ill [33, 43, 50, 51]. Between 2018 and 2021, adults traveling from Nigeria were diagnosed with monkeypox in Israel, the United Kingdom, Singapore, and the United States; however, all of them had a travel history to endemic regions of Central and West Africa [7, 46]. Compared with the past, monkeypox cases occurred suddenly and unexpectedly in several countries in May 2022, and these cases have no direct connection with areas that have experienced monkeypox for a long time, suggesting that monkeypox is much more likely to go out of Africa.

## Infectious sources and transmission routes of monkeypox

The host range and pathogenesis of MPXV are unknown. Rodents are likely to be potential hosts. In addition to rodents, nonhuman primates and humans can be reservoirs of infection [52, 53]. The occurrence of monkeypox in humans is more due to animal-to-human transmission than to limited human-to-human transmission [54, 55]. The main route of transmission is contact transmission. In Africa, where nutrition is poor, contact with live and dead animals through hunting and consumption of wild game or bush meat is a known risk factor for children. Children in countries outside Africa, however, were probably infected by MPKV through exposure to imported pet rodents [56]. While it is unclear where the current monkeypox cases originated, it is possible that through its evolution, MPXV increased its transmissibility among humans. Reports of women's cases herald children also in high-risk settings.

## Susceptible population

Humans are generally susceptible to monkeypox, with infection occurring predominantly in unvaccinated children, adults and immunocompromised individuals [57, 58].

## Drugs and vaccines

Although there is currently no standard-of-care treatment for monkeypox and it can only be managed through supportive care and symptomatic treatment, the emergence of smallpox antiviral drugs with poxvirus activity has been used against monkeypox [59]. Antiviral drugs currently used for smallpox mainly include tecovirimat (ST-246 or TPOXX) and brincidofovir, which were approved by the United States Food and Drug Administration (FDA) in 2018 and 2021, respectively, for the treatment of smallpox under the Animal Rule [60, 61]. In addition, both oral drugs have demonstrated efficacy against other orthopoxviruses (including monkeypox) in multiple animal models [62–64]. Tecovirimat and brincidofovir have been shown to be safe and well tolerated at the recommended human doses in human clinical safety trials [65]. Tecovirimat prevents the spread of the virus by inhibiting the function of the major envelope protein VP37, preventing the virus from leaving the infected cell. Tecovirimat is primarily indicated for the treatment of adults and pediatric patients weighing at least 13 kg [66, 67]. In comparison, Brincidofovir achieves its antiviral effect by integrating into the viral DNA, thereby inhibiting the action of viral DNA polymerase. Brincidofovir has stable absorption and can be used in very low birth weight infants and even neonates in emergency situations [60, 61, 68]. Additionally, other antiviral drugs, such as cidofovir and vaccinia immune globulin, if necessary, can be used in combination with brincidofovir and tecovirimat for the treatment of monkeypox disease [69] (Table 1). Notably, neither drug has been studied in human efficacy trials, particularly in the treatment of human monkeypox [70]. There is currently no effective treatment except for early vaccination after exposure to the virus.

**Table 1:** Summary of the treatments for monkeypox management

Treatments	Route	Mechanism of action	Dosing regimens	Applicability in children	Side effects
Tecovirimat (TPOXX, ST-246)	PO or IV	Inhibiting envelope protein VP37 of the orthopoxvirus	Pediatrics: if 13 kg to less than 25 kg: 200 mg twice daily for 14 d; if 25 kg to less than 40 kg: 400 mg twice daily for 14 d; if 40 kg or more: 600 mg twice daily for 14 d Adults: 600 mg twice daily for 14 d	Pediatric patients weighing at least 13 kg	The most common drug-related adverse events was headache
Cidofovir (vistide)	IV	Inhibiting the action of viral DNA polymerase	5 mg/kg once weekly for 2 wk, followed by 5 mg/kg IV once every other wk	Topical cidofovir has been successfully used to treat children with molluscum contagiosum or orf	Cidofovir is associated with dose-limiting nephrotoxicity, which is characterized by proteinuria followed by glucosuria, decreased bicarbonate, uric acid, and phosphate
Brincidofovir (CMX001, Tembexa)	PO	Inhibiting the action of viral DNA polymerase	Adult and pediatric patients weighing ≥ 10 kg to less than 48 kg: 4 mg/kg of the oral suspension once weekly for two doses; pediatrics weighing less than 10 kg: the dose is 6 mg/kg of the oral suspension once weekly for 2 doses Adults weighing ≥ 48 kg: 200 mg once weekly for two doses	Can be used in emergencies for low birth weight infants and neonates	May cause elevation of serum transaminases and serum bilirubin
Vaccinia immune globulin	IV	Passive immunity with antibodies obtained from the pooled human plasma of individuals immunized with the smallpox vaccine	6000 U/kg as soon as symptoms appears; may repeated based on severity of symptoms and response to treatment; 9000 U/kg may be considered if patient does not respond to initial dose	Effectiveness in treating monkeypox unknown	Headache, rigors, nausea, dizziness

*PO* peros (orally), *IV* intravenous

Furthermore, preexposure vaccination with smallpox vaccines has been shown to be protective in multiple animal models against a variety of OPXV challenges, and it has been estimated to provide 85% cross protection against monkeypox infection [17, 55]. Currently, the second-generation licensed smallpox vaccine ACAM2000 (a live‐attenuated replicating vaccine) and the third-generation vaccine JYNNEOS (a replication-deficient MVA vaccine) are two FDA‐approved vaccines that can prevent monkeypox. However, ACAM2000 is not suitable for immunocompromised individuals due to its high toxicity [71, 72]. The youngest reported case was a child with a parent who received ACAM2000; 11 days later, the child was exposed to a used dressing from the parent's vaccination site, and two days later, he/she developed a “pimple-like reaction above the left eyebrow”. Similarly, inadvertent transmission of ACAM2000 may occur, including vertical transmission from mother to child, which may be fatal to the fetus or newborn. Myopericarditis was a more frequent serious adverse event with ACAM2000^®^ than with JYNNEOS [69]. As such, it is only for use by military personnel and laboratory personnel working with OPXV in the United States but is not administered to the public [73]. The newly developed third-generation smallpox vaccine, modified vaccinia Ankara (MVA), is safe for immunocompromised individuals and has also been approved by the European Medicines Agency for the prevention of adults identified as at high risk of VARV and MPXV infection (18 years or older) smallpox and monkeypox, excluding children and infants [74]. Although MVA has demonstrated a good safety profile in phase III clinical trials, large-scale vaccination studies are still needed, and MVA has not been approved for use in the general population to prevent smallpox and monkeypox diseases. Additionally, its cost is also one of the factors to be considered [75]. In conclusion, ACAM2000 and MVA vaccines are still the best choice for postexposure prevention of monkeypox and smallpox.

## Conclusions

In the past 50 years, monkeypox has mainly occurred in the tropical rainforests of central and West Africa. Sporadic cases occur occasionally in countries outside Africa. Bunge et al. pointed out that in the early years (1970–1989), monkeypox was mainly a childhood disease, the median age of onset was four to five years, and 100% of the deaths occurred in children under ten years of age, while for the years 2000 to 2019, children less than ten years of age accounted for 37.5% of the deaths. Moreover, since 2000, the median age of monkeypox cases has increased from ten years in the first decade to 21 years in the second decade [46]. The reason for this age change may be that smallpox was eliminated in 1980. The conventional vaccine is no longer available to the public. It must be emphasized that although the mortality rate of monkeypox infection in children has changed to some extent, it still accounts for a high proportion of deaths. In a recent outbreak, as of August 29, 2022, more than forty-five thousand cases of monkeypox have been reported outside Africa. Genomic analysis results show that the MPXV strain associated with these outbreaks is the WA strain [76]. Early epidemiological reports of the initial cases indicated that the disease occurred mainly in MSM. For the recent monkeypox outbreak, however, the clinical presentation of monkeypox-related cases is mostly atypical and has been variable, and more analysis is still needed to determine the source of the infection. At present, some cases of community transmission, including in women and children, are being reported by some countries. This suggests that monkeypox is also more likely to spread among children. Additionally, MPXV has spread rapidly in a short period of time, causing widespread concern around the world. We can think of three possibilities that explain why MPXV is capable of cross-border spreading: (1) the virus mutates to increase the infectivity of the disease; (2) smallpox vaccination has been stopped; (3) human immunity to MPXV has been reduced, and this has greatly increased the risk of infection; and (4) changes in behavioral patterns of individuals have impacted the spread of the disease.

Despite the progress made in antiviral drugs against smallpox and monkeypox and vaccine development, other public health measures, such as monkeypox monitoring, case investigation and contact tracking, avoiding contact with animals or materials suspected of carrying the disease, using personal protective equipment and maintaining hand hygiene, are still the best measures to prevent and control monkeypox [77, 78]. In addition, the development of monkeypox/smallpox vaccines, monitoring, and MPXV detection technology are critical for the prevention and control of monkeypox. In countries where monkeypox cases are found, the epidemiology and transmission mode should be investigated as much as possible to control the transmission of MPXV in time.

## Data Availability

This manuscript has no associated data or the data will not be deposited (authors’ comment: this is a theoretical study and no experimental data).
